# The contrasting short‐term effects of COVID‐19 on dental care practices in the United States

**DOI:** 10.1002/cre2.398

**Published:** 2021-01-25

**Authors:** Zeid Diab, Nawar Shara, Jiling Chou, Alexander Libin, Alexander Kuhn, Cherae Farmer‐Dixon, Andrea Jackson, Bertram Hughes, Brian Laurence

**Affiliations:** ^1^ Department of Biostatistics and Biomedical Informatics MedStar Health Research Institute Hyattsville Maryland USA; ^2^ Health, Physical Education and Exercise Science Virginia Commonwealth University Richmond Virginia USA; ^3^ Department of Medicine Georgetown‐Howard Universities Center for Clinical and Translational Science Washington DC USA; ^4^ Office of the Dean Meharry Medical College Nashville Tennessee USA; ^5^ Office of the Dean Howard University College of Dentistry Washington DC USA; ^6^ President National Dental Association Foundation Gainesville Florida USA; ^7^ Department of Restorative Services Howard University College of Dentistry Washington DC USA

**Keywords:** African Americans, COVID‐19, dental care, ethnic groups, pandemic, racial disparities

## Abstract

**Objectives:**

The study utilized a cross‐sectional survey to determine the short‐term effects of the COVID‐19 pandemic on dental care practices. The authors hypothesized that the effects of the pandemic would indicate differences based on the ethnicity of the participating dentist.

**Materials and Methods:**

The survey was available online between June 1, 2020 and July 10, 2020, a period when many dental offices remained closed, and for the most part, unable to provide non‐emergency dental care. The link to the survey was made available to dentists through outreach to several national dental organizations. Descriptive statistics summarized the characteristics of the entire sample and Fisher's exact test was used to examine respondents' answers stratified by ethnicity using frequencies and percentages.

**Results:**

All ethnic groups reported decreased revenue and African American dentists were the least likely to report a decrease in revenue compared to White and Other ethnic groups (84.2%, 87.2% and 92.9%). African American dentists were the most likely to report willingness to contribute to a task force to address the new challenges resulting from COVID‐19 when compared to White and Other ethnic groups (46.4%, 18.8%, and 29.6%, respectively). African American dentists were more likely to indicate a need for a stronger connection to academic programs as compared to White or Other dentists in order to address current and future challenges (12.3%, 0.0%, and 9.1%).

**Conclusion:**

The COVID‐19 pandemic has affected dental practices differently, highlighting racial disparities, and strategies that factor in the race or ethnicity of the dentist and the communities in which they practice need to be considered to ensure that underserved communities receive needed resources.

## 1 INTRODUCTION

The COVID‐19 pandemic has challenged the healthcare industry and imposed an unprecedented impact on society. Communities of color have been impacted disproportionately exposing unsettling racial disparities and worsening disease outcomes on minority groups; particularly African Americans and Hispanics (Kullar et al., 2020). The Centers for Disease Control and Prevention (CDC) stated that “non‐Hispanic blacks, Hispanics, American Indians and Alaska Natives generally have the poorest oral health of any racial and ethnic groups in the United States,” (Centers for Disease Control and Prevention, 2020a) and adds that these same populations have a disproportionately higher incidence of COVID‐19–related infection and death (Centers for Disease Control and Prevention, 2020b).

The dental care community have been economically impacted as response to the COVID‐19 pandemic led to a temporary pause in many states on the provision of comprehensive dental care between March 2020–July 2020. This left a large sector of dental care professionals unable to treat patients or assist during uncertain and turbulent pandemic times. A recent study by the American Dental Association predicted a reduction of 37.4% in U.S. dental spending in 2020 as a result of the COVID‐19 pandemic's effect on dental practice revenues for the first half of 2020 (Nasseh & Vujicic, 2020). Dental health has far‐ranging impacts and dentists can fulfill important roles as part of the pandemic response team including participating in diagnosis, surveillance, education, risk communication, and notification.

We hypothesize that the effects of the pandemic on dental practice would differ based on the ethnicity of the participating dentists. Through the dissemination of a Dental Practice Pandemic Preparedness Questionnaire (DPPPQ), the aims of this just‐in‐time study were to explore the short‐term effect of the COVID‐19 pandemic on dental practices and to examine the willingness of dental providers to join a network of dentists to develop directives for early detection, prevention and treatment of COVID‐19 and future pandemics.

## 2 MATERIAL AND METHODS

Prior to its conduct, this project was reviewed and approved by the MedStar Health Research Institute Institutional Review Board (MHRI IRB) under federal wide assurance number FWA00000504. The MHRI IRB is located at 6525 Belcrest Rd. Ste. 700 Hyattsville, MD 20782 and can be contacted at 301‐560‐2912 or MHRI-ORIHelpDesk@MedStar.net. The board considered this project exempt and therefore did not require informed consent.

The DPPPQ was designed as a cross‐sectional survey aimed at collecting information from dentists on the impact of the COVID‐19 pandemic on different aspects of their practice and existing preparedness plans to face future pandemics. Dentists were asked to provide their demographic information and respond to questions about the effect COVID‐19 had on practice revenue and operations, ability to navigate through the pandemic, preparations for future challenges and willingness to join a network that could provide early notification of regions where COVID‐19 incidence may be increasing. The survey was available online between June 1, 2020 and July 10, 2020, a period when many dental offices remained closed and for the most part unable to provide non‐emergency dental care. The link to the survey was made available to dentists through outreach to several national dental organizations. We also shared the survey with the appropriate contact person for the dental society for each state in the United States. We specifically targeted national dental organizations that have higher numbers of minority dentists. The study protocol and the survey were approved by the Institutional Review Board at Medstar Health Research Institute.

A checklist of 24 questions with various response options were initially developed by the study team in collaboration with dental experts. In day 2 after the survey was sent out, the checklist was updated with additional questions based on respondents' feedback. The final checklist was completed by the subsequent participant (*N* = 183), excluding the first 10 participants who provided their feedback for the initial pool of questions. For the analysis phase, questions asking participating dentists to what extent they agree with the answer options presented, the responses “Very Much” and “Somewhat” were collapsed into one category, “Not Much” and “Not at all” into a second category, and the response “Neutral” created a separate category. For the analyses stratified by ethnicity, we collapsed dentists other than “African American” or “Whites” into one category defined as “Other” due to the small numbers in each of the remaining ethnic groups.

Descriptive statistics summarized the characteristics of the entire sample and Fisher's exact test was used to examine respondents' answers stratified by ethnicity using frequencies and percentages. All analyses were performed with R software with statistical significance set at *p* ≤ 0.05. Though our survey was disseminated nation‐wide, responses collected anonymously, and statistical analysis was blinded, the potential for self‐selection bias of those who completed the survey is possible.

## 3 RESULTS

A total of 193 dentists completed the DPPPQ, Table 1 shows that respondents were evenly split between males and females (48.4% vs. 49.5%). White dentists made up 46.0% of the respondents, African Americans were at 30.5%, followed by Asians or Pacific Islanders (11.2%), and Hispanics or Latinos were at 2.7%. More than 60% of the respondents were 50 years of age or older or in practice for more than 20 years. General dentists made up 61.1% of the respondents, oral and maxillo‐facial surgeons made up 7.3%, orthodontists, periodontists, and endodontists were at 5.8% respectively, and both Pediatrics Dentist and Prosthodontics composed 5.3% of the respondents, with the remaining 3.8% comprised of other dental specialties. Most of the respondents practiced in New York State (53.2%), followed by the District of Columbia (16.1%) and Maryland (10.2%). Dentists from Texas and Rhode Island combined made up 8.1% of the respondents with the few remaining participants from various other states.

**TABLE 1:** Demographic characteristics of study participants

Variable	*N* (%)
Gender	*N* = 186
Male	90 (48.4%)
Female	92 (49.5%)
Other	4 (2.1%)
Ethnicity	*N* = 187
African American	57 (30.5%)
White	86 (46.0%)
Hispanic or Latino	5 (2.7%)
Native American or American Indian	1 (0.5%)
Asian/Pacific Islander	21 (11.2%)
Prefer not to specify	13 (7.0%)
Other	4 (2.1%)
Age group	*N* = 185
25–39	35 (18.9%)
40–49	34 (18.4%)
50–59	46 (24.9%)
60>	70 (37.8%)
Years in practice	*N* = 189
<5	13 (6.9%)
5–10	22 (11.6%)
10–20	39 (20.6%)
>20	115 (60.8%)
Practice category	*N* = 178
Private practice/Dental Service Organization/Group Practice	160 (89.9%)
Community‐based or public health environment	6 (3.4%)
Hospital‐based environment	6 (3.4%)
Academic environment	19 (10.7%)
Other	1 (0.6%)
Practice type	*N* = 159
Individual private practice	100 (62.9%)
Group practice	58 (36.5%)
Dental Service Organization (DSO) member	3 (1.9%)
Non‐Federal Community Health Center	2 (1.3%)
Other	4 (2.5%)
Profession	*N* = 190
General Dentist	116 (61.1%)
Oral and Maxillo Facial Surgeons	14 (7.3%)
Periodontist	11 (5.8%)
Endodontist	11 (5.8%)
Orthodontist	11 (5.8%)
Pediatric Dentist	10 (5.3%)
Prosthodontist	10 (5.3%)
Other	7 (3.8%)
Location	*N* = 186
New York	99 (53.2%)
District of Columbia	30 (16.1%)
Maryland	19 (10.2%)
Rhode Island	8 (4.3%)
Texas	7 (3.8%)
Other	12.4 (7.0%)

Table 2 shows the responses to selected questions stratified by ethnicity. Compared to White and Other dentists, African American dentists were more likely to agree that dental administrators in the state where they practiced had adequately considered the views of the dental community during the pandemic (62.5%, 54.8%, and 31.7%, respectively). However, compared to African American dentists, White and Other dentists were more likely to indicate that they did not agree that the state administrators had made the best decisions for dentistry based on available information at the time (12.8%, 33.7%, and 39.0%, respectively). Regardless of ethnicity, respondents were similarly prepared to work remotely during the pandemic with African American, White and Others reporting 33.3%, 27.4%, and 29.3%, respectively. As expected, all ethnic groups indicated that their practice showed decreased revenue with the ethnic group classified as Other having the largest percentage of respondents indicating a decrease (92.9%) and African American respondents the lowest (84.2%).

**TABLE 2:** Responses to selected survey questions stratified by ethnicity

Selected survey questions	Black or African American *N* = 57	White *N* = 86	Other *N* = 44	Total *N* = 187	*p*‐value (Fisher exact test)
To what degree have the administrators in the state where you practice adequately considered the views of the dental community during this pandemic					0.049*
Very much/Somewhat	30/48 (62.5%)	46/84 (54.8%)	13/41 (31.7%)	89/173 (51.5%)	
Neutral	5/48 (10.4%)	7/84 (8.3%)	6/41 (14.6%)	18/173 (10.4%)	
Not much/Not at all	13/48 (27.1%)	30/84 (35.7%)	22/41 (53.7%)	65/173 (37.6%)	
Not applicable	0/48 (0.0%)	1/84 (1.2%)	0/41 (0.0%)	1/173 (0.6%)	
To what degree do you believe the dental community has been treated unnecessarily harshly with forced closures during this pandemic					0.669
Very much/Somewhat	18/48 (37.5%)	36/85 (42.4%)	23/42 (54.8%)	77/175 (44.0%)	
Neutral	10/48 (20.8%)	16/85 (18.8%)	7/42 (16.7%)	33/175 (18.9%)	
Not much/Not at all	19/48 (39.6%)	32/85 (37.7%)	11/42 (26.2%)	62/175 (35.4%)	
Not applicable	1/48 (2.1%)	1/85 (1.2%)	1/42 (2.4%)	3/175 (1.7%)	
To what degree do you believe the administrators in the state where you practice have made the best decisions for dentistry based on the information available at the time					0.007*
Very much/Somewhat	25/47 (53.2%)	46 (53.5%)	18/41 (43.9%)	89/174 (51.2%)	
Neutral	16/47 (34.0%)	10 (11.6%)	7/41 (17.1%)	33/174 (18.8%)	
Not much/Not at all	6/47 (12.8%)	29 (33.7%)	16/41 (39.0%)	51/174 (29.3%)	
Not applicable	0/47 (0.0%)	1 (1.2%)	0/41 (0.0%)	1/174 (0.6%)	
What degree of confidence do you have in your practice/organization's ability to navigate through the COVID‐19 pandemic					0.197
Very much/Somewhat	39/48 (81.3%)	65 (75.6%)	29/41 (70.7%)	133/175 (76.0%)	
Neutral	3/48 (6.3%)	12 (14.0%)	10/41 (24.4%)	25/175 (14.3%)	
Not much/Not at all	6/48 (12.5%)	8 (9.3%)	2/41 (4.9%)	16/175 (9.1%)	
Not applicable	0/48 (0.0%)	1 (1.2%)	0/41 (0.0%)	1/175 (0.6%)	
To what degree do you believe your practice/Organization is adequately prepared to work remotely					0.936
Very much/Somewhat	16/48 (33.3%)	23/84 (27.4%)	12/41 (29.3%)	51/173 (29.5%)	
Neutral	6/48 (12.5%)	11/84 (13.1%)	7/41 (17.1%)	24/173 (13.9%)	
Not much/Not at all	20/48 (41.7%)	42/84 (50.0%)	19/41 (46.3%)	81/173 (46.8%)	
Not applicable	6/48 (12.5%)	8/84 (9.5%)	3/41 (7.3%)	17/173 (9.8%)	
How has the financial standing in your practice been affected since the COVID‐19 pandemic?					0.101
No change	5 (8.8%)	1 (1.2%)	0/42 (0.0%)	6/185 (3.2%)	
Increased revenue	1 (1.8%)	0 (0.0%)	0/42 (0.0%)	1/185 (0.5%)	
Decreased revenue	48 (84.2%)	75 (87.2%)	39/42 (92.9%)	162/185 (87.6%)	
I do not know	1 (1.8%)	7 (8.1%)	3/42 (7.1%)	11/185 (6.0%)	
Other	2 (3.5%)	2 (2.3%)	0/42 (0.0%)	4/185 (2.2%)	
Not applicable	0 (0.00%)	1 (1.2%)	0/42 (0.0%)	1/185 (0.5%)	
Is your practice/organization re‐evaluating its strategy to be prepared in case of another pandemic outbreak as a direct result of the COVID‐19 pandemic?					0.180
Yes	28 (49.1%)	39 (45.4%)	17 (38.6%)	84 (44.9%)	
No	4 (7.0%)	10 (11.6%)	4 (9.1%)	18 (9.6%)	
Not sure	4 (7.0%)	13 (15.1%)	11 (25.0%)	28 (15.0%)	
I could use some guidance to devise a plan	21 (36.8%)	22 (25.6%)	10 (22.7%)	53 (28.3%)	
Other	0 (0.0%)	1 (1.2%)	2 (4.6%)	3 (1.6%)	
Not applicable	0 (0.0%)	1 (1.2%)	0 (0.00%)	1 (0.5%)	
Would you be willing to join a network that provides you with early notification of areas within the United States where the incidence of a pandemic (e.g., COVID‐19) has suddenly increased?					0.915
Yes	41/56 (73.2%)	59 (68.6%)	28 (63.6%)	128/186 (68.8%)	
No	5/56 (8.9%)	7 (8.1%)	5 (11.4%)	17/186 (9.1%)	
I am not sure	10/56 (17.7%)	18 (20.9%)	10 (22.7%)	38/186 (20.4%)	
Other	0/56 (0.0%)	2 (2.3%)	1 (2.3%)	3/186 (1.6%)	
Can you/your organization contribute to creating task forces to address the new challenges we face with COVID‐19?					0.006*
Yes	26/56 (46.4%)	16/85 (18.8%)	13 (29.6%)	55/185 (29.7%)	
No	8/56 (14.3%)	23/85 (27.1%)	5 (11.4%)	36/185 (19.5%)	
I am not sure	22/56 (39.3%)	46/85 (54.1%)	25 (56.8%)	93/185 (50.3%)	
Other	0/56 (0.0%)	0/85 (0.0%)	1 (2.3%)	1/185 (0.5%)	
Once the COVID‐19 pandemic is under control, how long would you project it would take for your practice activities to go back to normal?					0.452
1–4 months	22 (38.6%)	25/82 (30.5%)	11/43 (25.6%)	58/182 (31.9)	
5–8 months	12 (21.1%)	20/82 (24.4%)	4/43 (9.3%)	36/182 (19.8)	
9–12 months	9 (15.8%)	8/82 (9.8%)	10/43 (23.3%)	27/182 (14.8)	
>12 months	9 (15.8%)	22/82 (26.8%)	15/43 (34.9%)	46/182 (25.3)	
Never	5 (8.8%)	7/82 (8.5%)	3/43 (7.0%)	15/182 (8.2)	
What do dentists in your practice/organization need from businesses/industries to address the challenges we face both today and in the future?					0.010*
Stronger connection to academic programs	7 (12.3%)	0 (0.0%)	4 (9.1%)	11 (5.88%)	
Centralized source of vetted/useful information on how to prepare/handle this pandemic	38 (66.7%)	55 (64.0%)	30 (68.2%)	123 (65.78%)	
Clearer dialogue between researchers and industry practitioners	19 (33.3%)	29 (33.7%)	11 (25.0%)	59 (31.55%)	
Training tailored to handling pandemics	19 (33.3%)	18 (20.9%)	9 (20.5%)	46 (24.60%)	
I do not know	1 (1.8%)	4 (4.7%)	3 (6.8%)	8 (4.28%)	
Other	3 (5.3%)	4 (4.7%)	3 (6.8%)	10 (5.35%)	
Not applicable	0 (0.0%)	3 (3.5%)	1 (2.3%)	4 (2.14%)	

* *p*‐value <0.05.

Dentists from all ethnic groups indicated a strong willingness to join a network that provides early notification of a COVID‐19 incidence increase (73.2%, 68.6%, and 63.6%, respectively). African American dentists indicated a greater willingness to contribute to a task force created to address new challenges arising as a result of COVID‐19 compared to White and Other ethnic groups (46.4%, 18.8%, and 29.6%, respectively). Finally, African American dentists were more likely to indicate a need for a stronger connection to academic programs in order to address current and future challenges as compared to White or Other dentists (12.3%, 0.0%, and 9.1%) and were more likely to indicate a need for specific training in response to pandemics (33.3%, 20.9%, and 20.5%). Some respondents did not answer all questions, these were considered not applicable and excluded from the analysis. The total number of complete answers are reflected in Tables 1 and 2 above each question. There were 187 (96.9%) respondents who answered the ethnicity questions and of the 187 respondents approximately 92.5% to 100% answered the questions of interest.

## 4 DISCUSSION

This is one of the first studies to date that has compared the short‐term effects of the COVID‐19 pandemic on dental practices and examined racial disparities in context of the effects of the pandemic on the ethnicity of participating dentists. In accordance with the study hypothesis, we observed that the pandemic effects on dental practices differed by ethnicity. The survey was developed and deployed for a limited period of time as part of a larger initiative by our research group to develop a network of dentists who are willing to take an active role in preparation for pandemic events. The study finding will inform our interactive technology development that will display real‐time COVID‐19 infection rates based on population density and social determinants of health at the census block level. This just‐in‐time approach will allow dentists to take early action in the event of actual or anticipated future localized COVID‐19 or similar pandemic outbreaks, thus providing tools to better manage their patients including those who might be at higher risk given their demographic profiles.

The results of our study are similar to several studies showing the pandemic had unanticipated effects on dental practices (Al‐Khalifa et al., 2020; Chamorro‐Petronacci et al., 2020; Kinariwala et al., 2020; Tysiąc‐Miśta & Dziedzic, 2020). Studies in India, Spain and Poland, reported results that dentists were generally not prepared to provide patient care during the COVID‐19 pandemic and that the pandemic has had economic repercussions contributing to increased challenges in providing optimal dental care. Our study findings suggest that African American dentists were affected differently by COVID‐19 as compared to White and Other dentists in select regions in the United States. The results also show that a large number of participating dentists from all groups indicated a willingness to join a network for early notification of increased COVID‐19 incidence and that African American dentists, in particular, were more interested in developing stronger connections to academic programs to address current and future challenges.

A strength of our study is the high representation of dentists who self‐identified themselves as African American. In the United States, 3.7% of practicing dentists are of African descent (Solana, 2019), however African American dentists comprised 30.5% of our study population. African American dentists are significantly underrepresented among practicing dentists in the United States, yet they serve a disproportionate share of minority and underserved communities (Mertz et al., 2017). Another strength of the study is that we administered our survey during the period when dental offices were primarily closed throughout the United States, a unique period that may not occur again. Study limitations include the small sample size and the possibility that responding dentists may have been those more likely to have been adversely affected by the COVID‐19 pandemic. Several state dental representatives we contacted indicated an unwillingness to share the survey with members due to survey fatigue from other COVID‐19 surveys that had been shared with their members. Given the fact that our participants are from only a few states, any generalizations that apply the results of this study to the larger population of dentists practicing in the United States should be made with caution.

The COVID‐19 pandemic has affected dental practices differently and strategies that factor in the race or ethnicity of the dentist and the communities in which they practice need to be considered to ensure that underserved communities receive needed resources. This study highlights the impact of the recent pandemic on dental practices which deserves greater attention in order to shed light into the racial disparities of minorities including African American and Hispanic populations.

## CONFLICT OF INTEREST

The authors have no conflicts of interest relevant to this article to report.

## AUTHOR CONTRIBUTIONS

All authors contributed to the design of the project, the data collection process, drafting the manuscript and reviewing revisions of the draft.

## Data Availability

The data that support the findings of this study are available from the corresponding author upon reasonable request.
